# A retrospective study of postoperative targeted therapy in ALK-positive lung cancer

**DOI:** 10.1038/s41598-023-34397-0

**Published:** 2023-05-23

**Authors:** Bin Wang, Yang Song, Zhuo Chen, Xiaona Su, Xin Yang, Zhi Wei, Junxia Chen, Chuan Chen, Mengxia Li

**Affiliations:** 1 Department of Oncology, Daping Hospital, Army Medical University, No. 10, Changjiang Branch Road, Yuzhong District, Chongqing, 400042 China; 2 Department of Cell Biology and Genetics, Chongqing Medical University, Chongqing, 400016 China; 3 Department of Pathology, Daping Hospital, Army Medical University, Chongqing, 400042 China; 4 Information Section, Daping Hospital, Army Medical University, Chongqing, 400042 China

**Keywords:** Cancer, Diseases

## Abstract

In this study, we aim to investigate the therapeutic effect and safety of ALK inhibitor in ALK-positive lung cancer patients. 59 patients with ALK-positive lung cancer from August 2013 to August 2022 were retrospectively recruited. The basic information, pathological type, clinical stage and treatment strategy were collected. These patients were divided into two groups, including 29 patients of conventional adjuvant chemotherapy, and 30 cases of targeted therapy. The patients in the targeted therapy group underwent adjuvant targeted therapy with crizotinib for 2 years. The observation indicators include curative effects and adverse events. The disease-free survival (DFS) and overall survival (OS) were also analyzed. We analyzed the pathological stages after adjuvant chemotherapy and targeted therapy in lung cancer, no significant difference in the p stage N and T was found between the two therapeutic groups. However, the DFS events, DFS median time and OS median time showed significant improvement in the targeted therapy group when compared with adjuvant chemotherapy (all *P* < 0.05). Besides, the patients under both therapeutic regimens presented some adverse events, among them elevated aspartate transaminase/alanine aminotransferase was the most common adverse event in all the patients, followed by nausea and vomiting. Our study identified that crizotinib-based postoperative targeted therapy helps improve the prognosis of patients with ALK-positive lung cancer, confirming that postoperative targeted therapy can be considered an effective and feasible therapeutic alternative.

## Introduction

Lung cancer, the second most prevalent malignant tumor, is the leading cause of tumor-related deaths worldwide^1^. Among all the common subtypes of lung cancer, non-small cell lung cancer (NSCLC) accounts for 85% of all cases^2^. Most of the patients are at advanced stages once diagnosed due to the atypical manifestations, the 5-year overall survival (OS) rate for NSCLC is approximately 25%^3–5^. Several well-accepted guidelines recommended postoperative adjuvant chemotherapy for NSCLC patients at stage II–IIIA and IB with high-risk factors, however, the long-term prognosis is still not satisfactory^6–8^. Nowadays, molecular detection has become a mandatory approach for NSCLC management, helping guide diagnosis and treatment. Several gene mutations and translocations were found and incorporated in the NSCLC diagnostic standards, such as EGFR, MET and anaplastic lymphoma kinase (ALK)^9,10^.

Adjuvant treatment refers to the application of supplemental therapeutic approaches such as chemotherapy, radiation therapy, hormone therapy, and targeted therapy, aiming to eliminate potential residual cancer cells in the body after the operation and prevent tumor recurrence^11^. Adjuvant treatment is typically recommended for patients with early-stage NSCLC who have high-risk factors of recurrence such as larger tumor size, lymph node involvement, and genetic mutations^12^. Chemotherapy is commonly applied as an adjuvant treatment for NSCLC, consisting of a combination of medicine given in cycles^13^. In recent years, targeted therapy, such as the use of tyrosine kinase inhibitors, has been regarded as a promising treatment way for NSCLC patients with specific genetic mutations such as ALK^14^.

ALK is a transmembrane receptor tyrosine kinase that belongs to the insulin receptor superfamily^15^. ALK rearrangements in NSCLC were first reported in 2007, occurring in around 3–8% of overall NSCLC patients^16^. ALK-positive NSCLC is with confirmative driver genes and is highly sensitive to ALK inhibitors. To date, the U.S. Food and Drug Administration has approved several biological agents such as crizotinib, ceritinib and brigatinib as the first-line medicine for ALK-positive NSCLC^17^. It is reported that the 5-year OS rate for patients treated with next-generation ALK inhibitors such as Alectinib and lorlatinib in the first-line setting for ALK-positive NSCLC exceeds 60% in the ALEX study^18^. Due to their satisfactory efficacy, good safety and individualized features, ALK inhibitors are considered as the standard treatment for advanced NSCLC patients.

There are mainly three types of ALK gene mutation, including rearrangement, amplification, and point mutation^19^. ALK rearrangements are more commonly present in the younger cohort, non-smokers, and adenocarcinoma subgroups^20^. Patients with ALK-positive NSCLC present a higher postoperative recurrence, accompanied by shorter survival time, especially in patients at III stage^21^. Therefore, it is imperative to establish a more feasible and effective strategy for ALK-positive lung cancer patients. Till now, only a few case reports or studies with a relatively small number of subjects reported the postoperative targeted therapy for lung cancer patients at stages II–IIIA and IB. In this study, we performed a retrospective study to investigate the therapeutic effect, safety and optimal treatment regimen of ALK inhibitor in ALK-positive lung cancer patients.

## Materials and methods

### Subjects

A total of 2631 lung cancer patients who underwent the operation in Daping Hospital of Army Medical University from August 2013 to August 2022 were retrospectively recruited. Among them, 59 patients with lung cancer were detected as ALK-positive. All patients underwent full pathological mediastinal lymph node staging, and signed the informed consent. And this study was approved by the ethics committee of Daping Hospital of Army Medical University. The inclusion criteria for subjects collected in this study were listed as follows: (1) lung cancer diagnosed by pathology; (2) at tumor stage II–IIIA and IB according to the AJCC 8th edition; (3) confirmation of ALK-positive lung cancer; (4) complete surgical resection of the primary lung cancer; (5) no history of biological therapy or another experimental medication therapy; (6) no systemic infection requiring antibiotic treatment.

### Treatment methods

A total of 59 ALK-positive patients with lung cancer at stage pIB-IIIA who accepted lung tumor resection were classified into two groups, including 29 patients of conventional adjuvant chemotherapy, and 30 cases of targeted therapy. In the earlier years, more patients received chemotherapy as a form of treatment, whereas patients enrolled more recently were inclined to receive crizotinib owing to the advancement of ALK inhibitors. In the conventional adjuvant chemotherapy group, ALK-positive lung cancer patients with adenocarcinoma received pemetrexed plus cisplatin (PP regimen) treatment, patients with lung squamous cell carcinoma had paclitaxel plus cisplatin (TP regimen), and patients with small cell carcinoma received cisplatin plus etoposide (EP regimen) for 4 cycles. While the patients in the adjuvant chemotherapy group also received crizotinib treatment when the recurrence occurred. Another 30 cases in the targeted therapy group underwent adjuvant targeted therapy with crizotinib (250 mg bid) for 2 years after the operation until recurrence. If no recurrence occurred within a 2-year crizotinib treatment, then the medication was withdrawn. During this period, the computerized tomography (CT) examination was performed every 3–6 months to evaluate the potential recurrence.

### Observation indicators and evaluation criteria

The positive ALK expression was detected by Ventana-D5F3 assay using immunohistochemistry as previously described^22^. The indicators analyzed in this study include complete basic information about patients, pathological biopsy, clinical stages and risk factors. Then the curative effects, prognosis, as well as adverse events were analyzed, the specific indexes for prognosis assessment include disease-free survival (DFS) and OS. The DFS was referred to the period from the examination date to the date of disease recurrence or death (by any cause in the absence of recurrence). The OS was defined as the period from the examination time to death from any cause.

### Statistical analysis

The Shapiro–Wilk test was applied to verify the normal distribution of continuous variables. The continuous variables were represented as mean ± standard deviation if they were normally distributed, and the comparisons were conducted by *t*-test or ANOVA. If continuous variables were not normally distributed, they were expressed as medians (interquartile ranges), and the comparisons were performed using the Mann–Whitney U test or rank-sum test. Categorical variables were shown as counts (percent, %), and the comparisons between groups were performed using the chi-square test or Fisher exact test. Kaplan–Meier (KM) plots (two-sided 95% confidence interval) were used to analyze the total number of events and the median DFS and OS. The deadline of the present was November 1, 2022. *P*-value < 0.05 was considered statistically significant. All statistical analyses were analyzed by using R 4.2.1 (https://www.R-project.org/).

### Ethics approval

This study was performed in line with the principles of the Declaration of Helsinki. Approval was granted by the Ethics Committee of Daping Hospital of Army Medical University.

### Consent to participate

Informed consent was obtained from all individual participants included in the study.

## Results

### Characteristics of the patients

As shown in Table 1, a total of 59 patients were included in this study, with a median age of 53 years old. Among them, 31 patients (52.5%) were male and 28 patients (47.5%) were female. 88.1% of the patients had the pathological type of adenocarcinoma, 6.8% presented as squamous cell carcinoma, and the other 5.1% of patients showed small cell carcinoma. Regarding clinical stage, 3.4% of patients were at stage IB, 6.8% were at stage IIA, 18.6% were at stage IIB, and the other 71.2% were at stage IIIA. And 42.4% of patients had a history of smoking. Concerning the treatment methods, 30 patients (50.8%) received targeted therapy, and another 29 patients (49.2%) underwent adjuvant chemotherapy. There were no significant differences in age, ECOG ratio, pathological type, clinical stage and the smoking ratio between the two treatment regimens (all *P* > 0.05).

**Table 1:** Baseline demographic and clinical characteristics of patients with lung cancer.

	Level	Overall	Targeted therapy	Adjuvant chemotherapy	*P*-value
n (%)		59	30 (50.8)	29 (49.2)	
Age (years) (median [IQR])		53.00 [49.00, 63.50]	58.50 [49.50, 65.00]	53.00 [49.00, 62.00]	0.236
Gender (%)	Male	31 (52.5)	16 (53.3)	15 (51.7)	1
	Female	28 (47.5)	14 (46.7)	14 (48.3)	
ECOG (%)	0	53 (89.8)	26 (86.7)	27 (93.1)	0.699
	1	6 (10.2)	4 (13.3)	2 (6.9)	
Pathological type (%)	Squamous cell carcinoma	4 (6.8)	2 (6.7)	2 (6.9)	0.854
	Adenocarcinoma	52 (88.1)	26 (86.7)	26 (89.7)	
	Small cell carcinoma	3 (5.1)	2 (6.7)	1 (3.4)	
Clinical stage (%)	IB	2 (3.4)	2 (6.7)	0 (0.0)	0.538
	IIA	4 (6.8)	2 (6.7)	2 (6.9)	
	IIB	11 (18.6)	6 (20.0)	5 (17.2)	
	IIIA	42 (71.2)	20 (66.7)	22 (75.9)	
Smoke (%)	0	34 (57.6)	17 (56.7)	17 (58.6)	1
	1	25 (42.4)	13 (43.3)	12 (41.4)	

*ECOG* Eastern Cooperative Oncology Group.

### Pathological stages after adjuvant chemotherapy and targeted therapy in lung cancer

We analyzed the clinical stage to evaluate the pathological stages of lung cancer patients after being treated with adjuvant chemotherapy and targeted therapy. As shown in Table 2, no significant difference in the p stage N and Y (%) was found between the two therapeutic groups (all *P* > 0.05).

**Table 2:** Pathological stages after two different treatment regimens.

	Level	Overall	Targeted therapy	Adjuvant chemotherapy	*P*-value
n		59	30	29	
p stage N (%)	0	13 (22.0)	9 (30.0)	4 (13.8)	0.263
	1	6 (10.2)	2 (6.7)	4 (13.8)	
	2	40 (67.8)	19 (63.3)	21 (72.4)	
p stage T (%)	1	12 (20.3)	5 (16.7)	7 (24.1)	0.548
	2	40 (67.8)	20 (66.7)	20 (69.0)	
	3	5 (8.5)	4 (13.3)	1 (3.4)	
	4	2 (3.4)	1 (3.3)	1 (3.4)	

### DFS and OS after adjuvant chemotherapy and targeted therapy in lung cancer

**Figure 1:**
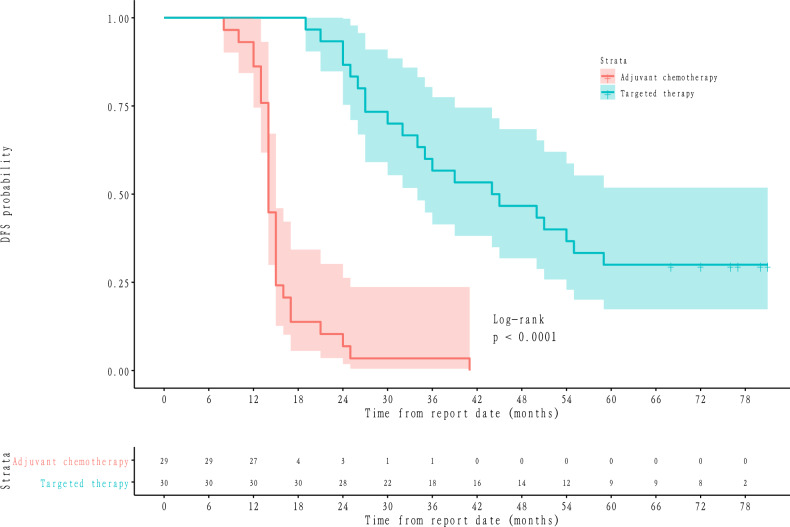
DFS probability between adjuvant chemotherapy and targeted therapy group shown by KM curves.

**Figure 2:**
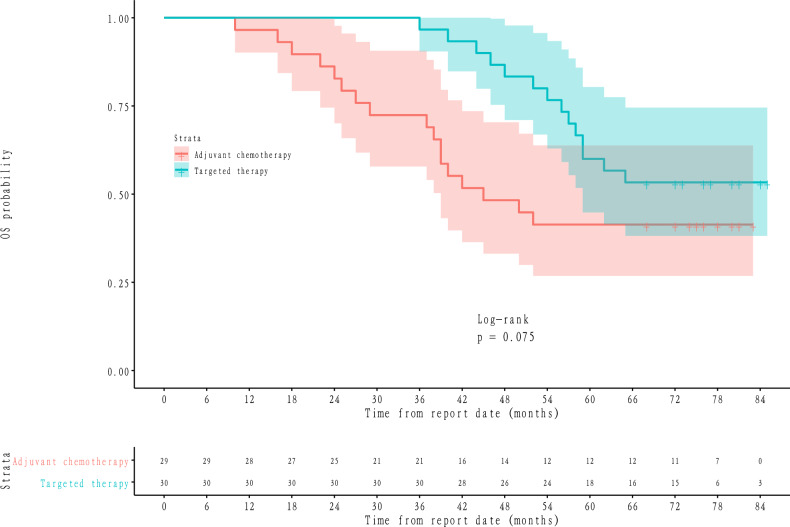
OS probability between adjuvant chemotherapy and targeted therapy group shown by KM curves.

**Figure 3:**
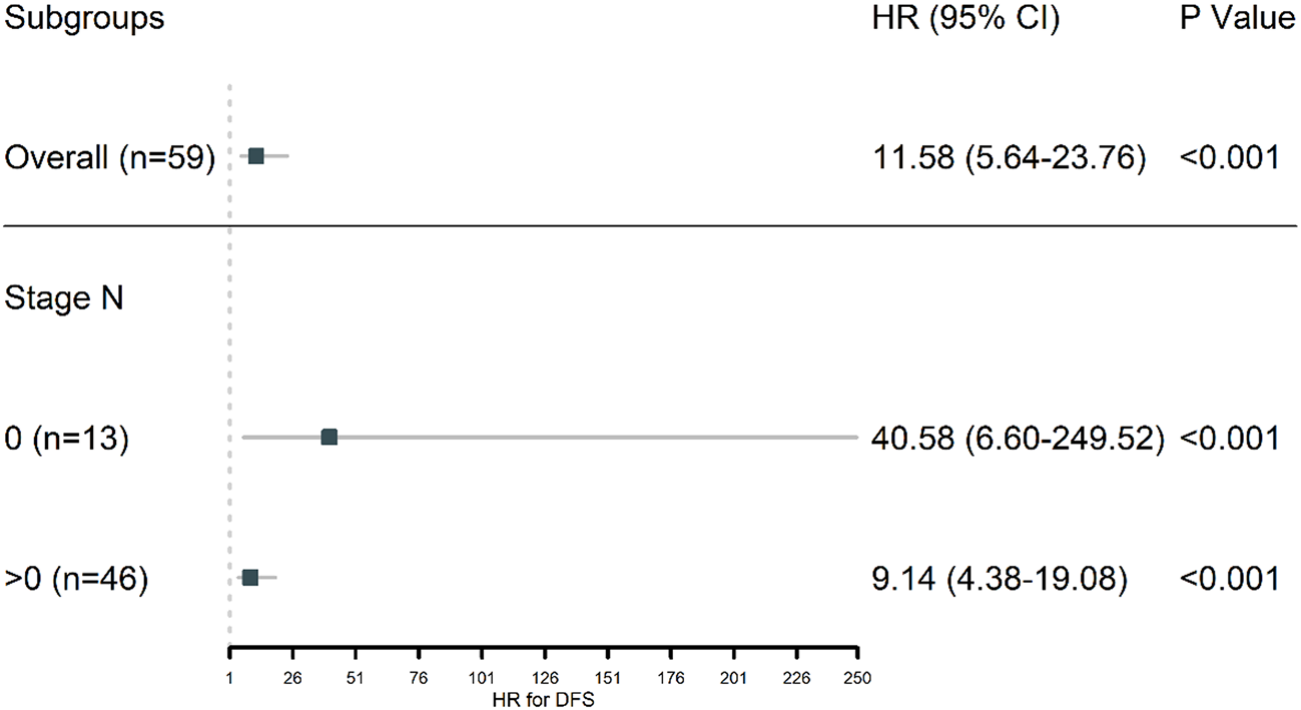
DFS of adjuvant chemotherapy and targeted therapy based on the general population and different N-stage levels.

In a total of 59 patients, the median follow-up time was 68 months. Of those, 50 patients (84.7%) achieved DFS events and 31 patients (52.5%) obtained OS events (Table 3). The median time of DFS and OS was 24 months and 62 months, respectively. It is shown that the number of DFS events, DFS median time and OS median time of patients were improved in the targeted therapy group compared with adjuvant chemotherapy (all *P* < 0.05), but there was no significant difference in the number of OS events. The KM curves of DFS and OS probability between adjuvant chemotherapy and targeted therapy group were shown in Figs. 1 and 2, and patients in the targeted therapy group represented a higher DFS and OS probability than adjuvant chemotherapy. Besides, we further analyzed the DFS of adjuvant chemotherapy and targeted therapy based on the general population and different N-stage levels. The results showed that the DFS rate of targeted therapy was significantly higher than that of adjuvant chemotherapy in both the general population and at different N stages (Fig. 3).

**Table 3:** DFS and OS in targeted therapy and adjuvant chemotherapy groups.

End points	Overall	Targeted therapy		Adjuvant chemotherapy	*P*-value
n	59		30	29	
Number of DFS events, (%)	50 (84.7)		21 (70.0)	29 (100.0)	0.004
DFS median time, month	24.00 [14.00, 44.50]		44.50 [27.75, 71.00]	14.00 [14.00, 15.00]	< 0.001
Number of OS events, (%)	31 (52.5)		14 (46.7)	17 (58.6)	0.51
OS median time, month	62.00 [41.00, 77.00]		70.00 [56.25, 77.00]	45.00 [29.00, 76.00]	0.021

*DFS* disease-free survival, *OS* overall survival.

### Adverse events of lung cancer patients after adjuvant chemotherapy and targeted therapy

Patients under both therapeutic regimens presented some adverse events, such as visual impairment, diarrhea, nausea and vomiting, pulmonary embolism, aspartate transaminase (AST)/alanine aminotransferase (ALT) elevation, neutropenia, lymphocytopenia, anemia, thrombocytopenia, peripheral edema, prolonged QT interval, interstitium lung disease/pneumonia, and hair loss (Table 4). Among them, elevated AST/ALT (71.2%) was the most common adverse event in all the patients, followed by nausea and vomiting (61%). When compared with adjuvant chemotherapy group, lung cancer patients in the targeted therapy group showed lower incidence rates of neutropenia, anemia and thrombocytopenia, while a higher incidence rate of vision impairment, QT prolongation and interstitial lung disease/pneumonia.

**Table 4:** Adverse events in targeted therapy and adjuvant chemotherapy groups.

	Level	Overall	Targeted therapy	Adjuvant chemotherapy	*P*-value
n		59	30	29	
Vision impairment (%)	0	46 (78.0)	17 (56.7)	29 (100.0)	< 0.001
	1	13 (22.0)	13 (43.3)	0 (0.0)	
Diarrhea (%)	0	27 (45.8)	16 (53.3)	11 (37.9)	0.355
	1	32 (54.2)	14 (46.7)	18 (62.1)	
Nausea and vomiting (%)	0	23 (39.0)	12 (40.0)	11 (37.9)	1
	1	36 (61.0)	18 (60.0)	18 (62.1)	
Pulmonary embolism (%)	0	56 (94.9)	27 (90.0)	29 (100.0)	0.248
	1	3 (5.1)	3 (10.0)	0 (0.0)	
AST/ALT elevation (%)	0	17 (28.8)	10 (33.3)	7 (24.1)	0.623
	1	42 (71.2)	20 (66.7)	22 (75.9)	
Neutropenia (%)	0	28 (47.5)	24 (80.0)	4 (13.8)	< 0.001
	1	31 (52.5)	6 (20.0)	25 (86.2)	
Lymphopenia (%)	0	27 (45.8)	13 (43.3)	14 (48.3)	0.905
	1	32 (54.2)	17 (56.7)	15 (51.7)	
Anemia (%)	0	44 (74.6)	27 (90.0)	17 (58.6)	0.014
	1	15 (25.4)	3 (10.0)	12 (41.4)	
Thrombocytopenia (%)	0	36 (61.0)	28 (93.3)	8 (27.6)	< 0.001
	1	23 (39.0)	2 (6.7)	21 (72.4)	
Peripheral edema (%)	0	44 (74.6)	19 (63.3)	25 (86.2)	0.086
	1	15 (25.4)	11 (36.7)	4 (13.8)	
Dysgeusia (%)	0	41 (69.5)	18 (60.0)	23 (79.3)	0.184
	1	18 (30.5)	12 (40.0)	6 (20.7)	
QT prolongation (%)	0	32 (54.2)	10 (33.3)	22 (75.9)	0.003
	1	27 (45.8)	20 (66.7)	7 (24.1)	
Interstitial lung disease/pneumonia (%)	0	51 (86.4)	22 (73.3)	29 (100.0)	0.009
	1	8 (13.6)	8 (26.7)	0 (0.0)	
Hair loss (%)	0	56 (94.9)	30 (100.0)	26 (89.7)	0.224
	1	3 (5.1)	0 (0.0)	3 (10.3)	

*AST* aspartate transaminase, *ALT* alanine aminotransferase.

## Discussion

The therapeutic landscape in ALK-positive NSCLC is rapidly developed in recent years, and lung cancers that harbor chromosomal rearrangements of ALK are highly sensitive to small-molecule tyrosine kinase inhibitors targeting at ALK^23^. Till now, specific therapeutic strategies and certain therapeutic effects of ALK inhibitors in ALK-positive lung cancer patients remain elusive. In the present study, we recruited a total of 59 patients with lung cancer and analyzed the efficacy and safety of postoperative targeted therapy in ALK-positive lung cancer. Although no significant difference in the p stage N and T was found between the two therapeutic groups in this study, the DFS events, DFS median time and OS median time were significantly improved in the targeted therapy group when compared with adjuvant chemotherapy. Besides, patients under both therapeutic regimens presented some adverse events, among them elevated AST/ALT was the most common adverse event in all the patients, followed by nausea and vomiting. Our patients in the targeted therapy group showed lower incidence rates of neutropenia, anemia and thrombocytopenia, but a higher incidence rate of vision impairment, QT prolongation and interstitial lung disease/pneumonia. This study helps confirm the effective and feasible therapeutic approach of postoperative targeted therapy in ALK-positive lung cancer, which is beneficial to improve prognosis in patients with lung cancer.

Adjuvant chemotherapy for NSCLC recommends the cisplatin-based combination medicine strategy, which usually begins as soon as the patient's physical condition is basically normal after the operation^24,25^. The suggested adjuvant chemotherapy generally starts at 4–6 weeks following surgery and not to exceed 3 months^26^. 4 cycles of postoperative adjuvant chemotherapy are often advised, additional cycles may not improve the patient's outcomes but may exacerbate adverse effects^27^. Of note, patients with stage IB-IIIA small-cell lung cancer are required to undergo surgical resection. Cisplatin-based combination medicine strategy is considered the standard postoperative chemotherapy regimen in patients with stage IB to stage IIIA lung cancer^28,29^. However, it is reported that only 5% of the lung cancer population acquires favorable effects, and the 5-year OS only increases by about 5%. Hence, more effective therapeutic methods need to be explored^30^.

ALK is a transmembrane tyrosine kinase receptor, composed of an extracellular domain, a transmembrane segment, and a cytoplasmic receptor kinase segment^31^. In NSCLC, ALK rearrangement is the most common type, accounting for 5–6% of patients, this mutation induces an oncogenic ALK tyrosine kinase that activates many downstream signaling pathways, thus leading to elevated cell proliferation and survival^32^. ALK inhibitors have emerged in recent years for postoperative targeted therapy in NSCLC patients, in which crizotinib, a small molecule ATP-competitive ALK inhibitor, is widely applied worldwide due to its favorable efficacy and relative safety^33,34^. In this study, we investigated the therapeutic effect and safety of crizotinib through comparing with postoperative adjuvant chemotherapy in ALK-positive lung cancer patients at stage II–IIIA and IB. Here, our baseline demographic showed a relatively higher smoker percentage compared with previous studies^35^, we speculated this difference may be related to the demographics, geographic location, or environmental exposures, besides, the present study is single-center research, which may lead to some bias of the patient's information.

In the current study, the treatment regimen was set as postoperative targeted therapy of crizotinib with a dose of 250 mg, bid for 2 years. Although the results showed no significant difference in the p stage N and T between the targeted therapy and adjuvant chemotherapy groups, we found that the number of DFS events, DFS median time and OS median time of patients were significantly improved in the targeted therapy group compared with adjuvant chemotherapy. Besides, DFS rate of targeted therapy was significantly higher than that of adjuvant chemotherapy in both the general population and at different N stages. Consistently, it is reported previously that more than 60% of patients with lung cancer show a satisfactory response to crizotinib, and the disease control rate is up to 90%^36^. Additionally, median progression-free survival exceeds 9 months, and the median OS could reach 75% after 1-year crizotinib treatment in ALK-positive lung cancer patients^37^. Shaw et al.^38^ conducted a global, randomized, phase 3 trial in 296 patients with advanced ALK-positive NSCLC treated with crizotinib, and the results showed that the percentage of patients who were alive without disease progression at 12 months was 39% in the crizotinib group, and the objective response occurred in 58% of crizotinib-treated cases, suggesting the beneficial role of targeted therapy in ALK-positive lung cancer patients.

In this study, we further evaluate the adverse events caused by postoperative targeted therapy or adjuvant chemotherapy. Among them, elevated AST/ALT was the most common adverse event in all the patients, followed by nausea and vomiting. We further found that the patients in the targeted therapy group had lower incidence rates of neutropenia, anemia and thrombocytopenia, but a higher incidence rate of vision impairment, QT prolongation and interstitial lung disease/pneumonia. The adverse events vary in two different treatment groups. Xin et al.^39^ demonstrated that 66.7% NSCLC patients suffered from crizotinib-induced liver toxicity and 11.9% progressed to severe hepatic toxicity. Solomon et al.^40^ also reported ALK inhibitor-related adverse events, including hypercholesterolemia (81%) and hypertriglyceridemia (60%). Thus, these adverse events caused by drug toxicities underscore the requirement to maintain a good safety profile through dose-limiting, especially when combining different treatments.

Some Phase III clinical trials investigating post-operative ALK inhibitor therapy in surgically resected ALK-positive lung cancer have been reported. For instance, ADJUVANT study is designed to assess the efficacy and safety of crizotinib as adjuvant therapy in ALK-positive NSCLC patients at stage IB, II, or IIIA after resection surgery, the trial is estimated to complete in 2023 (NCT01283516). Other ongoing Phase III clinical trial such as J-ALK study, aiming to explore the postoperative adjuvant therapy with crizotinib versus pemetrexed/cisplatin in ALK-positive patients with completely resected NSCLC (NCT02194738), it is estimated to complete in 2023.

There are some limitations in this study. First, due to the relatively long duration of patients’ enrollment (from 2013 to 2022), the regimen set here was the first-generation ALK inhibitor Crizotinib. Further therapeutic study with the latest generation of ALK inhibitor Alectinib is required. Next, this study is single-center and retrospective research with relatively small subject number, which may limit the interpretability of the findings. Moreover, patients treated with postoperative crizotinib treatment did not receive adjuvant chemotherapy, which may limit the optimal effectiveness of prolonging OS in surgically resected NSCLC.

## Conclusion

Collectively, crizotinib-based postoperative targeted therapy improves the outcomes of patients with ALK-positive lung cancer. However, attention still needs to be drawn to crizotinib-related adverse events, especially elevated AST/ALT and nausea and vomiting. Our study confirmed the effective and feasible therapeutic approach of postoperative targeted therapy in ALK-positive lung cancer, which is helpful to ameliorate the prognosis of patients. Further large-scale multi-center studies are worth performing to establish the therapeutic regimen of crizotinib-based postoperative targeted therapy in lung cancer patients.

## Data Availability

The datasets generated during and/or analysed during the current study are available from the corresponding author on reasonable request.

## Abbreviations

DFS: Disease-free survival
OS: Overall survival
NSCLC: Non-small cell lung cancer
ALK: Anaplastic lymphoma kinase
AST: Aspartate transaminase
ALT: Alanine aminotransferase

## References

[1] Schabath MB, Cote ML (2019). Cancer progress and priorities: Lung cancer. Cancer Epidemiol. Biomark. Prev..

[2] Relli V (2019). Abandoning the notion of non-small cell lung cancer. Trends Mol. Med..

[3] Golding B (2018). The function and therapeutic targeting of anaplastic lymphoma kinase (ALK) in non-small cell lung cancer (NSCLC). Mol. Cancer.

[4] Siegel RL (2021). Cancer statistics, 2021. CA Cancer J. Clin..

[5] Ettinger DS (2022). Non-small cell lung cancer, version 3.2022, NCCN clinical practice guidelines in oncology. J. Natl. Compr. Canc. Netw..

[6] Nakagawa K (2006). Randomised study of adjuvant chemotherapy for completely resected p-stage I–IIIA non-small cell lung cancer. Br. J. Cancer.

[7] He J (2015). Adjuvant chemotherapy for the completely resected stage IB nonsmall cell lung cancer: A systematic review and meta-analysis. Medicine (Baltimore).

[8] Wallerek S, Sørensen JB (2015). Biomarkers for efficacy of adjuvant chemotherapy following complete resection in NSCLC stages I–IIIA. Eur. Respir. Rev..

[9] Mack PC (2020). Spectrum of driver mutations and clinical impact of circulating tumor DNA analysis in non-small cell lung cancer: Analysis of over 8000 cases. Cancer.

[10] König, D., S. Savic Prince, & S.I. Rothschild. Targeted therapy in advanced and metastatic non-small cell lung cancer. An update on treatment of the most important actionable oncogenic driver alterations. *Cancers (Basel)*. **13**(4), 804 (2021). 10.3390/cancers13040804 PMC7918961 33671873

[11] Duma N, Santana-Davila R, Molina JR (2019). Non-small cell lung cancer: Epidemiology, screening, diagnosis, and treatment. Mayo Clin. Proc..

[12] Molina JR (2008). Non-small cell lung cancer: Epidemiology, risk factors, treatment, and survivorship. Mayo Clin. Proc..

[13] Pirker R (2020). Chemotherapy remains a cornerstone in the treatment of nonsmall cell lung cancer. Curr. Opin. Oncol..

[14] de Scordilli, M. *et al*. Targeted therapy and immunotherapy in early-stage non-small cell lung cancer: Current evidence and ongoing trials. *Int. J. Mol. Sci*. **23**(13), 7222 (2022). 10.3390/ijms23137222 PMC9266864 35806230

[15] Roskoski R (2013). Anaplastic lymphoma kinase (ALK): Structure, oncogenic activation, and pharmacological inhibition. Pharmacol. Res..

[16] Soda M (2007). Identification of the transforming EML4-ALK fusion gene in non-small-cell lung cancer. Nature.

[17] Lin YT (2021). Resistance profiles of anaplastic lymphoma kinase tyrosine kinase inhibitors in advanced non-small-cell lung cancer: A multicenter study using targeted next-generation sequencing. Eur. J. Cancer.

[18] Mok T (2020). Updated overall survival and final progression-free survival data for patients with treatment-naive advanced ALK-positive non-small-cell lung cancer in the ALEX study. Ann. Oncol..

[19] Zito Marino, F., *et al*. Unproductive effects of ALK gene amplification and copy number gain in non-small-cell lung cancer. ALK gene amplification and copy gain in NSCLC. *Int. J. Mol. Sci*. **21**(14), 4927 (2020). 10.3390/ijms21144927 PMC7404032 32664698

[20] Remon J (2021). Current treatment and future challenges in ROS1- and ALK-rearranged advanced non-small cell lung cancer. Cancer Treat Rev..

[21] Chaft JE (2018). Clinical outcomes of patients with resected, early-stage ALK-positive lung cancer. Lung Cancer.

[22] Shen Q (2015). Comparing four different ALK antibodies with manual immunohistochemistry (IHC) to screen for ALK-rearranged non-small cell lung cancer (NSCLC). Lung Cancer.

[23] Cooper AJ, Sequist LV, Lin JJ (2022). Third-generation EGFR and ALK inhibitors: Mechanisms of resistance and management. Nat. Rev. Clin. Oncol..

[24] Wang Y (2020). Effective treatment of lung adenocarcinoma harboring EGFR-activating mutation, T790M, and cis-C797S triple mutations by brigatinib and cetuximab combination therapy. J. Thorac. Oncol..

[25] Huang S (2020). Metformin reverses chemoresistance in non-small cell lung cancer via accelerating ubiquitination-mediated degradation of Nrf2. Transl. Lung Cancer Res..

[26] Kenmotsu H (2020). Randomized phase III study of pemetrexed plus cisplatin versus vinorelbine plus cisplatin for completely resected stage II to IIIA nonsquamous non-small-cell lung cancer. J. Clin. Oncol..

[27] Langer CJ (2017). Safety analyses of pemetrexed-cisplatin and pemetrexed maintenance therapies in patients with advanced non-squamous NSCLC: Retrospective analyses from 2 phase III studies. Clin. Lung Cancer.

[28] Douillard JY (2006). Adjuvant vinorelbine plus cisplatin versus observation in patients with completely resected stage IB-IIIA non-small-cell lung cancer (Adjuvant Navelbine International Trialist Association [ANITA]): A randomised controlled trial. Lancet Oncol..

[29] Zhong WZ (2018). Gefitinib versus vinorelbine plus cisplatin as adjuvant treatment for stage II-IIIA (N1–N2) EGFR-mutant NSCLC (ADJUVANT/CTONG1104): A randomised, open-label, phase 3 study. Lancet Oncol..

[30] Pignon JP (2008). Lung adjuvant cisplatin evaluation: A pooled analysis by the LACE Collaborative Group. J. Clin. Oncol..

[31] Reshetnyak AV (2021). Mechanism for the activation of the anaplastic lymphoma kinase receptor. Nature.

[32] Du X (2018). ALK-rearrangement in non-small-cell lung cancer (NSCLC). Thorac. Cancer.

[33] Molnar TF, Szipocs A, Szalai Z (2019). Neoadjuvant crizotinib for ALK re-arranged NSCLC?. J. Thorac. Oncol..

[34] Zhang C (2019). Neoadjuvant crizotinib in resectable locally advanced non-small cell lung cancer with ALK rearrangement. J. Thorac. Oncol..

[35] Cooper WA (2013). Molecular biology of lung cancer. J. Thorac. Dis..

[36] Kwak EL (2010). Anaplastic lymphoma kinase inhibition in non-small-cell lung cancer. N. Engl. J. Med..

[37] Camidge DR (2012). Activity and safety of crizotinib in patients with ALK-positive non-small-cell lung cancer: Updated results from a phase 1 study. Lancet Oncol..

[38] Shaw AT (2020). First-line lorlatinib or crizotinib in advanced ALK-positive lung cancer. N. Engl. J. Med..

[39] Xin S (2021). Impact of STAT1 polymorphisms on crizotinib-induced hepatotoxicity in ALK-positive non-small cell lung cancer patients. J. Cancer Res. Clin. Oncol..

[40] Solomon BJ (2018). Lorlatinib in patients with ALK-positive non-small-cell lung cancer: Results from a global phase 2 study. Lancet Oncol..

